# Bloodless Gynecological Surgery in Blood Products Refusing Patients: Experience of a Single Institution

**DOI:** 10.1089/whr.2023.0156

**Published:** 2024-04-18

**Authors:** Donatella Caserta, Flavia Costanzi, Maria Paola De Marco, Aris Raad Besharat, Christian Napoli, Maria Rosaria Aromatario, Stefano Palomba

**Affiliations:** ^1^ Gynecology Division, Department of Medical Surgical Sciences and Translational Medicine, Sant'Andrea University Hospital, Sapienza University of Rome, Rome, Italy.; ^2^ Department of Medical Surgical Sciences and Translational Medicine, Sant'Andrea University Hospital, Sapienza University of Rome, Rome, Italy.; ^3^ Department of Anatomical, Histological, Forensic Medicine and Orthopedic Science, Sant'Andrea University Hospital, Sapienza University of Rome, Rome, Italy.

**Keywords:** bloodless surgery, gynecological, Jehovah's Witness, iron, acid folic, vitamin B_12_

## Abstract

**Propose:**

This pilot study aimed to apply the central tenets of bloodless surgery and to analyze the effectiveness of specific preoperative, intraoperative, and postoperative strategies to minimize the risk for blood transfusion after gynecological surgery in a specific group of patients who refused blood products.

**Methods:**

A total of 83 patients undergoing gynecological surgery were included in the study. Forty-two patients received preoperatively oral iron, acid folic, and vitamin B_12_ supplementation in the 30 days before surgery, and 41 patients did not receive therapy.

**Results:**

No significant differences were found when comparing the two study groups. The implementation of all procedures to maintain a bloodless surgery has been helpful, in association with the other available procedures, in achieving optimal management and maintenance of hemoglobin levels, even in the most critical situations.

**Conclusion:**

In conclusion, implementing the bloodless approach as much as possible could guarantee the patient better and safer clinical and care management. Furthermore, well-designed research is required to clarify further the effects of bloodless surgery in gynecological patients.

## Introduction

The Italian National Blood Center promoted, in line with the Resolution of the World Health Assembly,^1^ an initiative aimed at systematizing innovative and more effective methods and tools to ensure the appropriateness of organizational and clinical management of blood resources. This initiative is identified in an innovative multiprofessional, multidisciplinary, and multimodal project that summarizes in the Anglo-Saxon definition of patient blood management (PBM).^2,3^ This strategy was already envisaged in the regional and national self-sufficiency program of the blood and its products of 2012.^4^ This program was aimed to give the definition and the implementation of “innovative and more effective methods and tools to ensure the appropriateness of the blood resource management, organizational and clinical.”

In November 2015, the Italian minister of health defined and implemented specific programs throughout the country, with particular reference to the preparation of the patient for scheduled surgical interventions. The PBM approach is based on the implementation of the three fundamental pillars: the optimization of erythropoiesis, the containment of blood loss, and the optimization of tolerance to anemia in the three essential phases of preoperative, operative, and postoperative patient management.^5–8^ A programmatic approach to PBM was associated with improved clinical outcomes, optimization of economic resources, and reduced transfusions.^9^

In the clinical practice, several concerns may be raised to refuse a transfusion of whole blood or other blood components, such as plasma, erythrocyte concentrates, leukocytes, and platelets. Those concerns include religious reasons (*i.e*., Jehovah's Witnesses), possible blood transfusion-related complications, an immunological incompatibility (rare groups and presence of alloimmunization), or unjustified fear. This problem cannot be overlooked in consideration of the need of specific and extensive informed consent for transfusion and its related medicolegal issues.^10^

Considering the specific group of Jehovah's Witnesses patients, it is also necessary to consider the pivotal role of the specific consensus that plays a fundamental role.^11^ Inviolability remains the more general principle of undoubted constitutional importance, by which the patient must be recognized as having the choice to be not treated even if such conduct exposes him to a high risk of life.^12^ Life belongs to the patient, who can decide whether or not to undergo a specific health treatment. The doctor should inform the patient of the consequences of the decision that exposes him/her to the risk of life.

It follows that any blood transfusion that is not permitted becomes unlawful because it violates the constitutional rules on freedom of conscience and the inconvertibility of individual health treatments. Considering the increasingly wide application of bloodless surgery in clinical practice, especially in cardiac and abdominal surgery,^13–16^ and due to the lack of specific data relating to gynecological surgery,^17–19^ this study aimed to analyze the effects of preoperatory management in specific blood products refusing patients scheduled for gynecological surgery. Therefore, our study reported our experiences related to the impact of an integrated strategy to minimize the risk of severe anemia in specific populations.^20^

## Materials and Methods

Blood products refusing patients scheduled for gynecological surgery were identified and screened before surgery between January 2016 and June 2022 at the gynecological division of the S. Andrea Hospital, Sapienza University of Rome, starting from digitalizing clinical data.

Inclusion criteria were age >18 years and signed refusal of blood products.

Exclusion criteria were previous surgery in the last year, iron deficiency without iron store replacement, myelodysplasia, myeloid cancers, ore pure red cell aplasia, uncontrolled hypertension, and history of thrombosis.

The study included 83 patients. All patients have expressed their willingness not to receive blood transfusions and products and signed a detailed informed consent.

All patients were subjected to a careful clinical evaluation and preoperative planning based on different points to search for risk factors and unrecognized pathologies.

- Anamnestic evaluation: The history of anemia, hereditary or acquired bleeding disorders, concomitant diseases, and careful assessment of the pharmacological history were evaluated.
- Investigate the manifestations of the disease materially associated with hemostatic dysfunctions (purpura, petechiae, ecchymosis, hepatomegaly, and splenomegaly).
- Specific laboratory tests: The risk of anemia with complete blood count and martial structures (serum ferritin receptor and transferrin receptor and estimation of bleeding risk by evaluation of coagulation factors and more detailed studies to identify coagulation disorder including specific tests for coagulation factors if risk factors) were assessed.

Our department developed a preoperative optimization protocol for patients refusing blood products, including oral iron and vitamin supplementation (folic acid and B_12_). The patients who did not perform or refused treatment were included in the control group (group 2). Therefore, patients were divided into two groups: 42 patients (group 1) in the 30 days before surgery were administered daily 1 tablet of oral iron (325 mg ferrous sulfate), 5 mg of folic acid, and vitamin B_12_ (methylcobalamin 1000 mcg). Group 2 (41 patients) did not receive any therapy. All included patients had a starting hemoglobin (Hb) between 10.8 and 14.3 g/dL.

The institutional review board (IRB) approval of the S. Andrea Hospital—Sapienza University of Rome was obtained and a careful informed consent was signed by each patient enrolled in the study protocol.

Statistical analysis was performed using SPSS version 26.0 (SPSS, Inc., Chicago, IL). Data are expressed as mean (±standard deviation) or as proportion (percentage, %). The changes in Hb levels were assessed with analysis of variance. Pearson chi-square test was used to assess the difference in proportions. A *p*-value <0.05 was considered statistically significant.

## Results

A total of 83 patients' afferent at the gynecology department of the hospital S. Andrea were included between January 2016 and June 2022. Preoperative patient characteristics are described in Table 1. All the blood products refusing patients were Jehovah's Witness.

**Table 1.** Patients' Characteristics

Patients' characteristics (***n***)	Total: Mean (±SD)/***n*** (%)	Group 1 ***n***:42	Group 2 ***n***:41	***p***
Age	51.78 ± 12.02 (30–82)	52.11 ± 13.9 (30–82)	51.91 ± 11.53 (34–82)	0.943
Parity				
0	20 (24.1%)	7 (16.6%)	13 (31.7%)	0.054
1	28 (33.7%)	12 (28.6%)	16 (39%)	
>2	35 (42.2%)	23 (54.8%)	12 (29.3%)	
Smoke				
Yes	44 (53%)	29 (69.1%)	15 (36.6%)	0.030
No	39 (47%)	13 (30.9%)	26 (63.4%)	
Diabetes				
No	65 (78.3%)	40 (95.2%)	15 (36.6%)	0.001
Yes	18(21.7%)	2 (4.7%)	16 (39%)	
Hypertension				
No	61 (73.5%)	25 (59.5%)	36 (87.8%)	0.076
Yes	22 (26.5%)	17 (40.4%)	5 (2.2%)	
Dyslipidemia				
No	65 (78.3%)	34 (80.9%)	31 (75.6%)	0.745
Yes	18 (21.7%)	8 (19%)	10 (24.4%)	
Previous cardiovascular event				
No	79 (95.2%)	38 (90.5%)	41 (100%)	0.270
Yes	4(4.8%)	4 (9.5%)	0	
Dysthyroid				
No	72 (86.7%)	33 (78.6%)	39 (16.6%)	0.057
Yes	11 (13.3%)	9 (21.4%)	2 (4.8%)	
Previous surgery	40 (48.2%)	18 (42.8%)	22 (53.6%)	
No				0.054
Abdominal surgery	26 (31.3%)	16 (38.1%)	10 (24.4%)	
No abdominal surgery	17 (15.7%)	7 (16.6%)	10 (24.4%)	
Symptomatology				
None	5 (6%)	4 (9.5%)	1 (2.4%)	0.316
Dysmenorrhea	2 (2.4%)	0	2 (4.8%)	
Pelvic pain	23 (27.7%)	13 (30.9%)	10 (24.4%)	
AUB	53 (63.9%)	25 (59.5%)	28 (16.6%)	
Cause of surgery				0.165
AUB	36 (43.4%)	14 (33.3%)	22 (53.6%)	
Fibromatosis	21 (25.1%)	11 (26.2%)	10 (24.4%)	
Ovarian cyst	10 (12%)	8 (19%)	2 (4.8%)	
Endometrial disease	10 (12%)	6 (14.3%)	4 (9.7%)	
Cancer	3 (3.6%)	1 (2.3%)	2 (4.8%)	
Miscarriage	2 (2.4%)	2 (4.7%)	0	
Endometriosis	1 (1.2%)	0	1 (2.4%)	
Surgery procedure				
Hysterectomy	22 (26.5%)	8 (19%)	14 (16.6%)	0.010
Myomectomy	15 (18,2%)	8 (19%)	7 (16.6%)	
Hysteroscopy	30 (36.1%)	14 (33.3%)	16 (39%)	
Interventions in the annex site	13 (15.6%)	7 (16.6%)	6 (16.6%)	
Vulvar surgery	2 (2.4%)	2 (4.7%)	0	
RCU	1 (1.2%)	1 (2.3%)	0	

AUB, abnormal uterine bleeding; SD, standard deviation.

In 43.4% of cases, patients were selected for surgical procedure for abnormal uterine bleeding, 25.1% for fibromatosis, 12% for benign adnexal disease, 3.6% for a tumor diagnosis, and 12% of cases for endometrial pathology (polyp, dysplasia) detected. The surgery procedure performed were 22 hysterectomies, 15 myomectomies, 30 hysteroscopies, 13 interventions in the annex site, 2 vulvar surgery, and 1 uterine revision. The main complications were one severe with acute bleeding, treated with uterine artery embolization, and five minor complications (three hyperpyrexia treated with paracetamol and two surgical site infections).

The gynecological division of S. Andrea Hospital managed preoperative optimization of the patient's Hb. The patients were divided into two groups. Forty-two of the patients received preoperative iron, folic acid, and vitamin B_12_ therapy in the 30 days before the surgical procedure (Group 1), whereas 41 received no therapy (group 2). The Hb value was assessed at the beginning of the study, the day before surgery, and after surgery.

Comparing group 1 and group 2 Hb presurgical values (13.7 ± 0.49 vs. 13.3 ± 0.28; *p* = 0.0768) and postsurgical values (13.3 ± 0.28 vs. 12.17 ± 0.57; *p* = 0.9453) and the values of the individual groups (group 1: 13.7 ± 0.49 vs. 13.3 ± 0.28; *p* = 0.4805; group 2: 13.3 ± 0.28 vs. 12.17 ± 0.57; *p* = 0.0790), no significant statistical difference was found (Table 2). No difference in the two study groups was found in Hb values before and after surgery after treatment.

**Table 2.** Pre- and Postoperative Comparison of Hemoglobin Values

	Mean (±SD)	***p***
Hb at start of the study	12.91 ± 0.88 (10.6–14.4)	
Hb pre (total population)	13.03 ± 0.86 (10.8–14.3)	
Hb post (total population)	12.04 ± 1.67 (5.4–14.3)	
Hb pre vs. Hb post (group 1)	13.7 ± 0.49(10.8–13.2) vs. 13.3 ± 0.28(5.4–14.0)	0.4805
Hb pre vs. Hb post (group 2)	13.3 ± 0.28 (12.6–14.1) vs. 12.17 ± 0.57 (9.8–10.3)	0.0790
Hb pre (group 1) vs. Hb pre (group 2)	13.7 ± 0.49(10.8–13.2) vs. 13.3 ± 0.28 (12.6–14.1)	0.0768
Hb post (group 1) vs. Hb post (group 2)	13.3 ± 0.28(5.4–14.0) vs. 12.17 ± 0.57 (9.8–10.3)	0.9453

Hb, hemoglobin.

Furthermore, no differences were found by stratifying the value by type of intervention. Comparing the pre- and postoperative Hb values in the two study groups, no statistically significant differences were found, stratifying for the different types of intervention such as hysterectomy (*p* = 0.915; *p* = 0.295), myomectomy (*p* = 0.902; *p* = 0.435), hysteroscopy (*p* = 0.932; *p* = 0.531), and interventions in the annex site (*p* = 0.773; *p* = 0.778) (Table 3).

**Table 3.** Pre- and Postoperative Comparison of Hemoglobin Values for Different Surgical Procedures

	Total population Mean (±SD)	Group 1 Mean (±SD)	Group 2 Mean (±SD)	***p***
Hysterectomy (*n*)	Tot: 22	8/22	14/22	
Hb pre	13.02 ± 0.85 (10.8–14.3)	13.23 ± 0.87 (11.8–14.3)	13.19 ± 0.83 (12.7–14.3)	0.915
Hb post	12.01 ± 1.41 (5.4–14.2)	11.60 ± 2.60 (5.4–13.7)	12.40 ± 0.84 (11–14.2)	0.295
Myomectomy (*n*)	*n* = 15	8/15	7/15	
Hb pre	12.67 ± 0.80 (10.8–14.2)	12.63 ± 0.80 (10.8–13.8)	12.69 ± 1.06 (11.3–14.3)	0.902
Hb post	10.76 ± 1.80 (5.5–13.7)	11.81 ± 2.09 (9.8–13.2)	11.14 ± 0.71 (10.8–14.2)	0.435
Hysteroscopy (*n*)	*n* = 30	14/30	16/30	
Hb pre	13.22 ± 0.72 (11.8–14.3)	13.26 ± 0.72 (11.8–14.3)	13.18 ± 0.74 (12.1–14.3)	0.932
Hb post	12.56 ± 1.03 (10.8–14.3)	13.53 ± 1.02 (10.8–14)	12.59 ± 1.06 (10.8–14.2)	0.531
Interventions in the annex site (*n*)	*n* = 13	4/13	9/13	
Hb pre	12.88 ± 1.11 (10.8–13.8)	12.72 ± 1.20 (10.8–13.8)	12.33 ± 1.05 (10.8–13.9)	0.773
Hb post	12.14 ± 1.43 (9.8–13.2)	11.96 ± 1.51 (9.8–13.2)	11.20 ± 1.78 (9.8–13.3)	0.776

Of the 83 patients who had “bloodless” gynecological surgery at S. Andrea University between January 2016 and June 2022, none died before hospital discharge or within the 90-day follow-up.

Depending on the clinical case, all procedures were implemented during the surgical procedure to minimize the risk of bleeding. Acute normovolemic modulation (depending on Hb values), performed in 8 interventions, intraoperative recovery of blood performed in 14 interventions, the use of minimally invasive surgical techniques in 42 surgical procedure, and topical hemostatic agents and electrocoagulation elements performed in all the intervention, has allowed, in most cases, to control and minimize bleeding.

Relating to the complications detected, a single severe complication after a multiple myomectomy has been found. Severe anemia at the end of the surgical procedure was managed through uterine vessel embolization by an interventional radiology unit, determining a resolution of bleeding. The Hb values improved from 5.4 g/dL on the postoperative day to 7.8 g/dL on registration (on the 12th day). The average number of days of hospitalization was 1 day for minor surgery performed in day surgery (hysteroscopy, vulvar surgery, and uterine revision).

Major surgery was 3.1 days for a hysterectomy, 3.4 days for a myomectomy, and 2.1 days for interventions in the annex site. Concerning the side effects reported after preoperative therapy, four patients reported mild abdominal or stomach pain, two registered nausea, and one notified constipation. No patients stopped therapy during the study period.

## Discussion

This retrospective study reported no difference in the two patient groups analysed. No significant difference in haemoglobin levels between patients who performed pre-operative therapy with iron, folic acid and vitamin B compared with patients who did not receive treatment. We considered a number of generic, medical, and surgical variables. In 2021, ∼2.9 million blood transfusions were performed in Italy (data from the ministry of health).

In addition, the blood transfusion or its components may pose a risk to the recipient related to the appearance of both immediate and late undesirable reactions. Because the possible risks are not always predictable, transfusion therapy should be prescribed only after carefully analysing the risks and benefits associated with it.^21^ The transfusion of blood cells could be considered, from an immunological point of view, a real transplant with the possibility of rejection reactions is also very harmful to the recipient.^22^

It should also be considered as, for a growing subset of patients, such procedures are not an option due to personal preferences, religious beliefs, or biological conditions such as advanced sickle cell anemia with antibodies. In such circumstances, doctors may turn to a bloodless approach that avoids the need for transfusions. Surprisingly, such approaches, which use a variety of clinical and laboratory methods, have been shown to reduce blood loss and accelerate recovery, minimize infection, and reduce the length of hospital stays.^23,24^ A vital aspect of bloodless preoperative care is identifying and treating any pre-existing anemia well before the surgical procedure.

Methods range from oral iron therapy to intravenous iron treatment combined with erythropoiesis-stimulating agents. In our study group, no state of severe anemia was found in the preoperative period. These data can determine why there are no significant differences between treated and nontreated patients. These results follow the current literature meta-analytical data concerning iron oral administration,^25^ especially confirming how a bloodless approach in an adequately prepared and selected population determines a similar performance in the study groups.

It is essential to underline that our pilot study has some limitations, including a small study sample and a more significant risk of a type II error. In addition, a comparison with a control group that does not refuse blood products will be a target of future evaluations.

Concerning informed consent, it should be noted that in the current Italian legal system, the patient has the choice not to receive treatment (Article 32 of the Constitutional Charter, Article 35 of the Code of Medical Ethics, and Article 5 of the 1997 Oviedo Convention on the Rights of the Union and Biomedicine).

There is a conflict between subjective assumptions and health. The doctor must treat and respect the patient's decision (without any legislation, administrative or judicial authority, being able to change things).

Great attention must be paid to the hierarchical order of the sources of law between the right of self-determination of the patient for the refusal to care (right to let himself die, not to want death) and the duties incumbent on the doctor who must take action and do, according to science and conscience, everything possible to safeguard the patient's health. The refusal to treatment (in this case, blood transfusions) must be the subject of a clearly expressed, unequivocal, current, informed, and understood manifestation. These considerations must also draw attention to how this increases the need to implement all possible alternatives to rejected procedures.

In conclusion, there is a lack of high-quality evidence for managing patients who refuse blood products and perform gynecological surgery.^17–19^ Blood transfusion is a fundamental and pivotal means of managing a hemorrhagic patient. However, considering its risks, costs, and the risk of limited blood supply, knowing and implementing the PBM approach as much as possible are essential to guarantee the patient better and safer clinical and care management, as underlined by our pilot study. Further well-designed randomized studies are required to provide further definitive information about the benefits and potential harms of pre-, intra-, and postsurgical management of gynecological patients.

## Abbreviations Used

AUB: abnormal uterine bleeding
Hb: hemoglobin
IRB: institutional review board
PBM: patient blood management
